# Research on the Utilization of Carbonated Red Mud in Sustainable Construction Materials (Paving Stones)

**DOI:** 10.3390/ma19091883

**Published:** 2026-05-02

**Authors:** Augustin Voinea, Gheorghe Voicu, Mihail Savaniu, Adrian Lazarescu, Paula Tudor

**Affiliations:** 1 Department of Biotechnical Systems, National University of Science and Technology Politehnica Bucharest, Splaiul Independenței 313, S.6, 060042 Bucharest, Romania; augustin.voinea@stud.isb.upb.ro; 2 Faculty of Mechanical Engineering and Robotics in Construction, Technical University of Civil Engineering of Bucharest, Bd. Lacul Tei 124, 020396 Bucharest, Romania; mihai.savaniu@utcb.ro; 3 Cluj-Napoca Branch, National Institute for Research and Development in Construction, Urbanism and Sustainable Territorial Development INCD URBAN-INCERC, 400524 Cluj-Napoca, Romania; adrian.lazarescu@incerc-cluj.ro; 4 Department of Entrepreneurship and Management, National University of Science and Technology Politehnica Bucharest, Splaiul Independenței 313, S.6, 060042 Bucharest, Romania; paula.voicu@upb.ro

**Keywords:** red mud, accelerated carbonation, ALUM Tulcea, construction materials, circular economy, compressive strength, geopolymers, sustainability, carbon footprint, sodium leaching, sustainable pavements

## Abstract

The management of red mud (bauxite residue, RM) is critical for environmental protection due to its high alkalinity (pH 12.5–13.0). The study investigates the valorization of RM from the ALUM Tulcea unit (Romania) through accelerated wet carbonation processes (L/S ratio 4:1) and its integration into sustainable construction materials (paving stones). The results indicate a reduction the pH to a stable level of 8.6 in 240 min, a process validated by the formation of new mineral phases (calcite and dawsonite) that stabilize the residual sodium. For the optimized recipe S2 (20% RM, 12% cement, 48% fly ash), an average compressive strength of 33.8 MPa (class T5 according to SR EN 1338:2004) and a low water absorption of 4.12% (Class B) were obtained. Durability tests confirmed superior freeze–thaw resistance (mass loss 0.58 kg/m^2^) and sodium (Na^+^) leaching below 2.1 mg/L, well below EU limits. In the case of alkaline activated geopolymers (NaOH 8 M), the strength reached 38.5 MPa. The study demonstrates that carbonated RM can reduce the carbon footprint by 20–56% and production costs by up to 43%, providing a viable circular economy solution in line with EU 2030 targets.

## 1. Introduction

Investigations on the neutralization of red mud from alumina plants have been carried out quite extensively internationally. The study proposed by us makes additional investigations on the use of a protocol at accelerated wet carbonation (slurry carbonation) of red mud in 240 min, in order to transform it from a waste considered hazardous into an inert material.

The production of alumina from bauxite via the Bayer process is associated with the generation of red mud as the primary waste material, also known as “bauxite residue”. Depending on the quality of the processed raw material, approximately 1–2.5 tons of red mud are generated for every ton of alumina produced. The treatment and disposal of this residue represent a major operation in an alumina refinery (55–65% of the processed bauxite). Red mud is the residue resulting from the Bayer process of alumina extraction from bauxite through bauxite digestion with sodium hydroxide at high temperature and pressure [1]. The disposal and neutralization of red mud can lead to its utilization in the construction field (geopolymers, clay materials, cements, ceramics, fired and unfired building materials, the concrete industry), as well as in metal recovery (iron, titanium, aluminum, alkali, rare earths), or its use as a coagulant, adsorbent, and catalyst in soil remediation. Additionally, it can be utilized in environmental pollution control (wastewater treatment, absorption, and purification of acidic waste gases) [2].

The composition of red mud is mainly iron oxides ranging from 30 to 60%, which gives it its red color, aluminum oxides (Al_2_O_3_), about 10–20%, silica (SiO_2_) in a wide range of 3–50%, and varying percentages of calcium oxide (CaO) and sodium oxide (Na_2_O) which gives it its alkalinity. The exact percentages vary depending on the origin of the bauxite ore and the production process (such as the Bayer process), but it also contains smaller amounts of titanium dioxide (TiO_2_) and trace elements [3].

Iron oxides (Fe_2_O_3_) give red mud its red color.

Equation (1) shows why the resulting red mud has an extremely high pH. The presence of excess caustic soda (NaOH), visible on the left side of the equation, is why our research focuses on the subsequent carbonation process for neutralization.

Al_2_O_3_ + H_2_O + 2NaOH → 2NaAlO_2_ + (x + 1) H_2_O, (1)

The relationship represents the fundamental chemical equation by which bauxite is treated with sodium hydroxide (NaOH) at high temperature and pressure to extract alumina.

In his work, Cablik [4] indicates an Fe_2_O_3_ percentage of 48.5% and silica and Al_2_O_3_ contents of approximately 11.53% and 14.14%, respectively. The average density of dry RM is about 3.05 g/cm^3^.

The extraction of gibbsite from bauxite is rapid and occurs within minutes. The red mud generated in the process is sticky and has an average particle size, generally below 10 µm (with some exceptions of particles larger than 20 µm). It is composed of iron, titanium and silica from the parent ore, together with other minor constituents. It is alkaline, thixotropic and has a high specific surface area, between 13 and 16 m^2^/g, with a true density of 3.30 g/cm^3^.

Red mud is not a particularly toxic material, and the US Environmental Protection Agency (EPA) does not classify it as a hazardous waste [5], but the treatment and disposal of red mud is an important operation and can represent 30–50% of the operations in an alumina refinery.

Because bauxite is treated with sodium hydroxide, the red mud is very caustic, with a pH between 10.5 and 12.5. Bauxite rock has a specific gravity of 2.6–3.5 kg/m^3^, being an amorphous or clayey substance of pink color, but if it has a lower iron content, it can be whitish, but with the increase of iron content, it becomes reddish-brown [6].

Red mud has been found to have a high sorption capacity, making it effective for removing contaminants, such as cadmium, from soil and water. A major limitation to the use of available technologies for the use of red mud is the lack of cost–benefit analyses, given that there is high transportation costs associated with transferring red mud from disposal sites to the point of application [7].

Chemical analysis reveals that red mud contains silica, aluminum, iron, calcium, titanium, as well as a number of minor constituents, such as Na, K, Cr, V, Ni, Ba, Cu, Mn, Pb, and Zn [8].

Modern dewatering techniques feature semi-dry disposal methods to a solid’s concentration of 50–60% before pipeline transport. At the disposal site, the red mud is spread and allowed us to consolidate and dry in layers, following the method of thickened dumping and dry stacking [5].

The recovery and reuse of red mud from artificial ponds/lakes where it has been deposited requires drying, and then production of construction materials [9], production of coloring agents for painting works, toned paper in the pulp and paper industry, iron ore sinters and pellets in ferrous metallurgy or improvement of soil structure, as well as micro-fertilizer and pesticide neutralizer in agriculture.

An initial material without pretreatment, containing 42.2% Al_2_O_3_ and 14.2% Fe_2_O_3_ can be transformed into a product with 92.6% Al_2_O_3_ and 2% Fe_2_O_3_ with a recovery of 72%, which used in the Bayer process can lead to a substantial reduction in the amount of red mud.

After neutralization, mixtures of red mud and local clay can be used as land covers, in the rehabilitation of waste dumps, the terracing of roads and dams, can lead to the growth of vegetation, preventing erosion [5]. Many authors propose processing to obtain bricks or other building materials as a possible use for their red mud by treating sulfuric acid or using other methods.

Used as a pigment for painting concrete composites, the results presented by Cablik [4] show that RM is suitable for this. Samples taken at thicknesses below 2 µm, values required for pigments, and analyzed after 28 days of maturing, showed adequate resistance and ecological compatibility with the environment, especially if a previous calcination at 650 °C is performed [10].

Red mud sewage with red mud has also been used to produce artificial soil mixtures for the reclamation of degraded areas [11].

Treatment of red mud with citric acid and calcium nitrate (at reaction temperatures of 25–90 °C of 30–240 min led to reduced sodium content, low pH, increased particle size and reduced bioavailability of heavy metals. Mixed with natural soil in ratios, it has been shown to improve the physicochemical properties of red clay soil. Red mud soil planting experiments achieved high germination rates, results confirming the feasibility of modified red mud as a plant growth medium [12].

The authors of [13] investigated the performance of red mud on the strength and durability of concrete in which cement was replaced by 10%, 20% and 30% red mud (mass units). Based on the results obtained, the strength properties of concrete increased. The surface resistivity value is slightly higher compared to conventional concrete. The higher the surface resistivity value, the lower the conductivity property of concrete.

The average optimum pH for adsorption of the main anionic pollutants is 8.42 ± 1.13 (arsenite), 3.73 ± 0.68 (arsenate), 3.50 ± 2.38 (phosphate), 4.43 ± 1.04 (fluoride), and 3.80 ± 1.54 (chromate) [14].

So, there are three main aspects of using red mud: as a raw material mainly in the construction industry, as a source of valuable components such as rare earths and metals, especially iron, respectively in environmental applications such as wastewater treatment, soil remediation, etc. [15].

Table 1 and Table 2 show typical values of red mud for its physical and mechanical properties [3,4,5,16].

The use of RM pre-calcined at 600 °C improves the density and corrosion resistance of concrete due to its high alkalinity [17,18], while an addition of 20% RM optimizes durability and decreases water absorption [19]. At the same time, RM can be used as a pigment for concrete composites [4,10], in the manufacture of bricks, geopolymers, ceramic glazes and fillers [5,14,20].

In the field of environmental protection, RM can be used as an efficient adsorbent and flocculants for the removal of heavy metals (Cd, Zn, Pb), anions (phosphates, fluorides) and organic substances from wastewater [2,7,8,13]. In agriculture, RM modified with citric acid and calcium nitrate improves the structure of clay soils, reducing the bioavailability of heavy metals and promoting germination (≥84%) for species such as *Ulmus pumila* or *Populus alba* [11,12].

Tan et al. [21] and Yang et al. [22] demonstrates that the use of red mud in alkaline mixtures for semi-flexible pavements significantly reduces energy consumption and environmental impact, confirming their ecological safety.

Zhang et al. (2025) [23] proposes a new strategy for transforming red mud into lightweight aggregates and specialized cement for green concrete production, assessing sustainability through carbon footprint and life cycle cost analysis.

Wu et al. (2025) [24] states that extending the pre-cure duration significantly attenuating the obstruction of CO_2_ diffusion channels induced by NaHCO_3_, increasing the depth of carbonation, and shorter pre-cure exacerbates the loss of geopolymer strength during carbonation. Post-carbonation curing enhances geopolymer strength by increasing the CO_2_-derived product, carbonation modifies geopolymer products, increasing porosity.

Budige and colab. (2026) [25] analyze the use of slurry mixed with fly ash for masonry units, highlighting advantages in thermal insulation and cost reduction for affordable housing.

Padhan and Paul [26] conduct a comprehensive analysis of the physical and chemical properties of red mud from alumina production, review methods for recovering critical minerals from red mud, and evaluate the reuse of red mud in cement, brick, ceramic, and catalyst applications.

Zakira and colab. (2023) [27] synthesize a high strength geopolymer with industrial waste, through which they obtain a high initial strength using 50% red mud, also investigating the effect of mixing procedures and molar ratios on the microstructure.

In Romania, ALUM Tulcea in collaboration with various research institutes in the country (National Research and Development Institute for Pedology, Agrochemistry and Environmental Protection—ICPA Bucharest, Pitești-Albota Agricultural Research and Development Station—SCDA, Technical University of Cluj-Napoca) continues to identify solutions for transforming red mud into a product for commercial use, after it was investigated as an adjuvant for soil fertilization, or as an addition to the preparation of organic and chemical fertilizers in various experimental variants, increasing the alkalinity of the soil, or of the values of its chemical properties. The application of red mud on acidic soils is effective in preventing phosphorus leaching, improving soil acidity and, especially, reducing the presence of heavy metals in the soil through their ionic adsorption on the surface of red mud particles.

The use of red mud (bauxite residue resulting from alumina production) in the manufacture of paving stones represents an innovative solution for the management of industrial waste, with significant economic, structural and ecological benefits. It is highly alkaline and difficult to store, occupying significant land areas. Its integration into paving stones reduces the volume of industrial waste and the risk of soil and water contamination. Studies show that the addition of an optimized amount of red mud can improve the compressive strength and durability of paving stones, due to the high content of metal oxides (especially iron and aluminum). To make paving stones, it is necessary to improve the mechanical properties through the intensity of chemical hardening reactions. The paving stones produced develop a lower global warming potential, production costs are lower and the dependence on traditional raw materials, such as sand and natural aggregates, is reduced. At the same time, red mud can provide paving stones with natural color shades and resistance to freeze–thaw cycles are improved.

Our study investigates the valorization of red mud from the ALUM Tulcea unit (Tulcea, Romania) through accelerated carbonation processes and its integration into sustainable construction materials (paving stones). By integrating red mud in proportions of 10–35%, resistance class T5 (≥30 MPa) is achieved, according to the SR EN 1338:2004 [28] standard, which makes these pavers suitable for pedestrian and light traffic (e.g., supermarket parking lots). Road geopolymers using 35–40% alkaline activated red mud, eliminating Portland cement, achieved the highest strength (38.5 MPa) and reduced CO_2_ emissions by up to 56%.

## 2. Materials and Methods

### 2.1. Raw Materials and Characterization

The raw material used in this study was red mud (bauxite residue) supplied by the ALUM Tulcea refinery (Tulcea, Romania). The red mud samples were taken directly from the warehouse, presenting a high initial humidity (150–250%) and a strongly alkaline character, with a pH between 12.5 and 13.0.

The main chemical composition, determined by XRF, showed a high content of oxides: Fe_2_O_3_ (40–50%)—responsible for the red color; Al_2_O_3_ (18–20%); SiO_2_ (10–15%); and Na_2_O (8–12%); but other oxides were also present, such as CaO (2–10%)—for ALUM Tulcea (5–6%); TiO_2_ (2–0%)—for ALUM Tulcea (2–4%); and K_2_O below 5%. The red mud presented a critical alkalinity (pH 12.5–13.0) and a very narrow particle size distribution, with an average diameter d_50_ = 4.8 μm, which gives it a high specific surface area (18.4 m^2^/g) and superior pozzolanic reactivity This allows the material to act as an active micro-filler in the cementitious matrix. The comparative values for the chemical composition of RM from Alum Tulcea are presented in Table 3.

The high content of Fe_2_O_3_ and the presence of Na_2_O in the Tulcea red mud justify the need for prior carbonation to ensure the chemical stability of the cementitious matrix. Chemical analysis was performed to determine the oxide ratio (Fe_2_O_3_, Al_2_O_3_, TiO_2_, Na_2_O).

In addition to major oxides, red mud may contain traces of transition metals (Cr, Ni, V, Mn, Cu, Zn, Pb), rare elements (Sc, Y, La, Ce, Nd, etc.), anions and soluble substances (in leachate) (Na^+^, Cl^−^, SO_4_^2−^, F). These components may be important in the context of toxicity assessment and environmental protection.

Values of 5–15% define a stable but weak material for a highway foundation, but for pavements and urban developments, a substrate with CBR > 15% is ideal.

The high fine fraction (80% < 8 μm) and the significant plasticity index underscore the necessity for carbonation to improve workability and structural stability.

The red slurry from ALUM Tulcea presents a very fine grain size (d_50_ = 4.8 μm), with an extremely plastic character indicated by the high Atterberg limits (LL up to 300%). This colloidal structure, corroborated with an electrical conductivity of up to 10 mS/cm and the presence of rare elements such as Sc and La (0.1–0.5%), imposes the need for chemical stabilization by carbonation before use as a binder.

For the preparation of sustainable mixtures, the following complementary materials were also used: Portland cement (CEM I 42.5R), fly ash (from Mintia, Romania) or granulated slag (from Alro Slatina, Slatina, Romania) as pozzolanic materials, as well as aggregates (sand 0–4 mm) and superplasticizer additives for workability regulation.

CEM I 42.5R is a type of Portland cement commonly used in construction, with a minimum compressive strength of 42.5 MPa after 28 days. The “R” indicates that the cement has a high initial strength, hardening much faster in the first days (2–7 days) compared to the standard version (N—Normal).

As an “R” cement, it compensates for the tendency of red mud to sometimes slow down the setting of the material (when the pH is not properly controlled). Because it is CEM I (without other industrial additives), it allows researchers to observe exactly how the red mud interacts with the clinker, without the results being influenced by other components (such as slag already present in other types of cement). It is ideal for higher class pavers (T5–T7), ensuring that the threshold strength required for handling and traffic is quickly reached.

The paper analyzes four scenarios S1, S2, S3 and S4 which refer to specific mix recipes for construction materials (pavers and geopolymers).

Fly ash (from the Mintia Power Plant) is the main material for compensating the plasticity of the slurry, being a pozzolanic material rich in silica (SiO_2_) and alumina (A_2_O_3_). It acts by providing nucleation points for cement hydration, accelerating the formation of the C-S-H gel. It is composed of fine, mostly spherical particles, which improve the workability (flowability) of the mixture and is added to the S2 recipe to allow the achievement of the T5 strength class and reduce efflorescence to below 1%.

Red mud (from Alro Slatina) is used as a densifying additive in scenarios S1 and S3, acting as an active filler that increases abrasion resistance (according to the Böhme test, reaching Class I). It has a rough/irregular texture (unlike spherical ash), which helps to increase the mechanical stability of the pavers and achieve a resistance suitable for local roads.

Granulated blast furnace slag (GBFS) appears as a central element in scenario S4 (Geopolymer), where it completely replaces cement. It is a latent hydraulic binder, which means that it hardens rapidly under the action of the alkaline activators (NaOH) used in the study. It also favors the formation of C-A-S-H gels, which fill the voids left by the red mud particles, reducing porosity by up to 66%.

A high-performance polycarboxylate (PCE) superplasticizer was added to the analyzed recipes. The choice of this additive is justified by three critical needs of the project: it maintains a low Water/Binder (W/L) ratio; it has an extremely fine grain size (d_50_ = 4.8 μm) and a large specific surface area (15–40 m^2^/g), which tends to “absorb” water quickly. The additive is necessary to ensure particle dispersion and to prevent self-dehydration of the mixture.

The very small particle size (d_50_ = 4.8 μm) and the reactivity of the aluminum and silicon phases transform this waste into a densifying agent for construction materials.

### 2.2. Testing Methods

The determination methods for the characterization of red mud are divided into chemical, mineralogical and physico-mechanical analyses.

Chemical and mineralogical analyses refer to the determination of the major chemical composition (metal oxides) by X-ray fluorescence, respectively X-ray diffraction for the identification of mineral phases (hematite, boehmite, gibbsite) and new minerals appearing after carbonation (calcite, dawsonite), SEM/EDS for the analysis of morphology and fine particles, ICP-MS for the detection of heavy metals (Cr, As), respectively titrimetry (with 0.1 N HCl) for the determination of total alkalinity.

The analyses were carried out at the National Institute for Research and Development for Chemistry and Petrochemistry—ICECHIM Bucharest to highlight the modifications in the texture and morphology of the surface of the red mud particles before and after chemical treatment and thermal conditioning. A SEM microscope model FEI Quanta 200 (Thermo Fisher Scientific, Brno, Czech Republic), distributed in Romania by the Ronexprim company (Bucharest, Romania), equipped with an EDS (Energy Dispersive X-ray Spectroscopy) detector for elemental analysis was used. For ICP-MS analyses, equipment distributed by the AgilRom company (Agillent Technologies, Seoul, Republic of Korea) was used.

Physical properties analysis (SR EN/ISO Standards) refers to water content, by steaming at 105 °C (SR EN 1097-5:2008/ASTM D2216), [29,30] solid density determined with a water or helium pycnometer (SR EN ISO 11508) [31], sample granulometry, determined by sedimentation (hydrometer) and sieving (SR EN ISO 17892-4) [32], specific surface area of the red mud by the BET method (nitrogen adsorption), respectively pH and conductivity, for which a System 914 pH/Conductometer (Metrohm, Herisau, Switzerland) for high-precision electrochemical measurements are used (SR EN 13037/SR EN 13038) [33,34].

European Standard SR EN 1097-5 establishes the reference method for the determination of the total free water content (surface moisture plus accessible pore water) in aggregate samples. ASTM D2216 is the international standard for soil analysis in geotechnical engineering. The results are essential for compaction control, calculation of the plasticity index and other geotechnical analyses. Within SR EN ISO 11508, the material must be oven dried to constant mass, usually at a temperature of (105 ± 2) °C. The value obtained is essential for the calculation of soil porosity and pore volume, when used together with bulk density.

The technical criteria and operational parameters used for the red mud carbonation process targeted a pH reduction from extreme alkaline values (12.5–13.0) to values between 8.5 and 9.0, as well as a total alkalinity reduction of over 80%. The efficiency of the process was monitored by specific gas consumption estimated at 70–85 g/kg dry red mud.

To achieve these criteria, the suspension concentration had a liquid/solid ratio of 4:1 (with 20% solids), bubbling with pure CO_2_ or combustion gas (15–20% CO_2_) at a flow rate of 1–2 L/min. Agitation was maintained at 300–400 rpm to ensure gas dispersion. Reaction time was 30–120 min for wet carbonation (rapid pH drop in the first 30 min), respectively 4 h for complete stabilization at pH 8.6 (tests performed at ALUM Tulcea).

The mineralogical indicators (XRD) of post-process validation were the appearance of calcite (CaCO_3_) and dawsonite (NaAlCO_3_(OH)_2_), concomitantly with the disappearance of soluble sodium phases. The chemical indicators monitored were the decrease in Na_2_O content below the 3% threshold and sodium leaching below 5 mg/L (EU limit). Among the mechanical indicators, the increase in undrained shear strength from 5–20 kPa to 30–80 kPa immediately after the process was monitored.

These values were not chosen randomly, but represent an optimum between chemical efficiency and physical feasibility of the mixture, being based on recent references [35,36] and pilot tests carried out on the Tulcea RM.

The L/S ratio = 4:1 (20% suspension) facilitates optimal diffusion of CO_2_ into the liquid phase. At higher solids concentrations, viscosity increases, reducing the reaction rate. The RM from Tulcea is very fine, and this ratio, together with the addition of sodium hexametaphosphate (0.5 g/L), ensures uniform dispersion, preventing the formation of hard aggregates.

For RM, studies (including those with samples from ALUM Tulcea) show similar structures: particles between 10 and 100 μm, with homogeneous but dense distribution, and main phases of Fe-oxides. For the Tulcea dump, the microstructure is very similar to those at global level, since the Bayer process is standard.

**Figure 1:**
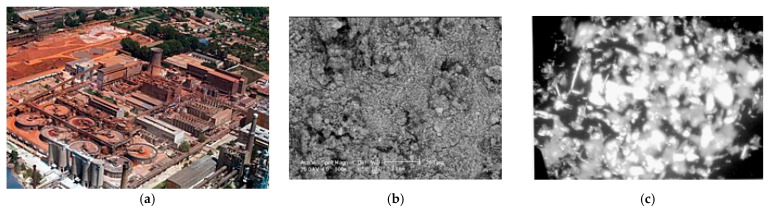
Aerial image of the ALUM Tulcea factory (**a**); SEM analyze for RM (1:100) (**b**); SEM analyze for raw RM Tulcea (1:2000) (**c**), [37,38].

Figure 1 presents the aerial image of the ALUM Tulcea factory along with SEM aspects of the RM from this factory.

Research shows that this ratio results in a rapid decrease in pH (from 12.8 to 10 in the first 15–30 min), facilitating the rapid neutralization of free NaOH.

The rotation speed was calibrated to handle the high density of the red mud particles (containing heavy iron oxides): 300 rpm was used for 10 min for the initial homogenization of the red mud powder in distilled water, ensuring wetting of all particles without introducing too much air; 400 rpm is the critical speed to keep the fine particles in suspension and to increase the contact surface between the bubbled CO_2_ bubbles and the liquid. A lower speed would allow sedimentation of the Fe_2_O_3_-rich material, while a much higher speed in the 3 L reactor could lead to the formation of a vortex that was too deep, reducing the residence time of the gas in the suspension.

When determining the mechanical properties, the Casagrande apparatus and the rolling method (SR EN ISO 17892-12) [39] were used for the Atterberg limits, respectively a Proctor hammer and die (SR EN ISO 13286-2) [40] for compaction tests, but also a direct shear apparatus or triaxial cell (SR EN ISO 17892-8/9/10) [41,42,43] or a laboratory press (ASTM D2166) [44] for specific compression tests on pavements according to SR EN 1338.

The determination of the plasticity index (PI) for RM ALUM Tulcea was carried out by standardized laboratory methods, using the difference between the Atterberg limits, defined by the relationship:

PI = LL − PL,(2)

where LL (yield limit) was determined by the Casagrande method, PL (plasticity limit) was determined by the 3 mm roller method, according to SR EN ISO 17892-12 standards. For a very high IP the water retention capacity is high, the material showing a high sensitivity to humidity variations. According to the standard procedure, the Casagrande apparatus, for determining LL, is used to find the water content at which a groove made in the sample closes over a distance of 12 mm after 25 blows. To determine PL, the roll is manually manipulated on small portions of material until they begin to crack exactly when they reach a diameter of 3 mm.

When drying samples (by oven method, at 105 °C) for determinations (including Atteberg limits), Romanian RM tends to form very hard aggregates, which is why a preliminary dispersion was made before analysis and a chemical dispersant (sodium hexametaphosphate) was used to obtain precise results on the fine fraction.

For RM from ALUM Tulcea, the plasticity index has a value of PI ≈ 16–20%, which places the material in the category of soils with medium plasticity, being similar to a dusty clay.

The values resulting from laboratory analyses on samples taken directly from the Tulcea dump are presented in Table 4.

In contrast to typical values reported in the literature (Table 1), the red mud from ALUM Tulcea exhibits a d_50_ of 4.8 μm. This unique structural feature allows the optimization of the recipes presented in Table 4, ensuring excellent leaching stability (0.3 mg/L Na^+^) and superior mechanical strengths. The superior performance of the S2 recipe (33.8 MPa) is directly correlated with the micro-filler property of RM Tulcea (d_50_), which allows a matrix densification inaccessible to materials with standard granulometry (>15 μm).

### 2.3. Mix Design and Sample Preparation

The neutralization process was carried out by a wet carbonation method (slurry carbonation) to reduce the pH of the slurry from 12.8 to values of approximately 8.6, ensuring chemical stability and improving mechanical properties, in accordance with [26,27]. The procedural steps included the preparation of the suspension, using a liquid/solid (L/S) ratio of 4:1 (2 L distilled water to 500 g dry slurry), homogenization of the suspension by continuous mechanical stirring at 300–400 rpm in a 3 L glass reactor.

Pure gaseous CO_2_ (typically 99.9%) (or 15% CO_2_/85% air mixture) was injected into the suspension at a constant flow rate of 1–2 L/min under ambient pressure (1 atm) and temperature (20–25 °C). Real-time monitoring of pH, electrical conductivity (EC), and temperature was performed at 5 min intervals to ensure process stability until the target threshold of pH < 9 was achieved [27,28].

To evaluate the mechanical performance of the pavers according to SR EN 1338:2004, four mix scenarios (S1–S4) were tested using carbonated red mud (pH 8.6), all optimized in 2025. In scenarios (S1/S2), CEM I 42.5R cement was replaced in a proportion of 10–20%, with the addition of fly ash (from Mintia) or slag (from Alro Slatina), while for scenario (S4) a cement-free mix of 35% carbonated red mud (pH 8.6), and 65% granulated blast furnace slag (Alro Slatina) mixed with fly ash (Mintia) (50% slag + 50% fly ash, the mixture being alkaline activated with a solution of NaOH (8 M) and Na_2_SiO_3_.

For scenario S3 (with the production of pavers for medium traffic) 25% carbonated red mud (pH 8.6), 10% CEM I 42.5R cement, 45% granulated slag (Alro Slatina) or fly ash (Mintia) (50% slag + 50% fly ash), 20% sand 0–4 mm, 1.5% superplasticizer were used.

For the composite cement, RM calcination at 800 °C, wet mixing (water/binder ratio 0.3–0.4) and vibration casting of the pavers were performed, while for the geopolymers (without cement), alkaline activators (NaOH, Na_2_SiO_3_) and heat treatment at 60–80 °C for 24 h were used. The ceramic blocks were then obtained by pressing at 10–20 MPa and firing in a furnace at temperatures of 900–1100 °C.

The conditioning of the paving samples included 28 days of maturation in a humid chamber at a temperature of 20 ± 2 °C and relative humidity RH > 95%. The paving stones were then immersed in water at 20 ± 5 °C before testing, and the surfaces were flattened (capping) with calcium sulphate mortar (max. thickness 1.5 mm) to ensure flatness, in accordance with SR EN 1338:2004.

For batch approval, a set of 10 whole pavers was subjected to compression, while 8 half pavers were used for the water absorption and freeze–thaw test. For other tests, results are usually averages obtained from 3 to 5 replicates per mix.

For casting into pavers, the water/binder ratio (W/L) was maintained between 0.3 and 0.4, with the use of a superplasticizer additive (1.2–1.5%) to ensure processing.

The compressive strength (UCS) was determined on 200 × 100 × 80 mm pavers after 28 days of curing in a humid chamber (RH > 95%), using a universal press Controls Automax (3000 kN) with a loading rate of 0.25 MPa/s, at a temperature of 20 ± 2 °C.

Further tests were carried out for water absorption, freeze–thaw resistance (28 cycles) and abrasion resistance (Böhme Method) to test the durability of the pavers and microstructural analysis was performed. The mineralogical evolution (i.e., the formation of calcite and dawsonite) was evaluated by X-ray diffraction (XRD) analyses.

## 3. Results

### 3.1. Efficiency of the Carbonation Process and Chemical Stabilization

The efficiency of the carbonation process was evaluated by continuously monitoring the pH of the red mud suspension (L/S 4:1) over a period of 240 min. The results indicate an important reduction in alkalinity, and the process can be divided into two distinct stages.

Stage 1 of rapid neutralization (from 0 to 30 min) showed an immediate decrease in pH from 12.8 to 10.2, due to the direct reaction of CO_2_ with free sodium hydroxide (NaOH) in the liquid phase.

The second stage, stabilization and precipitation, took place from 30 to 240 min, as follows: in the intervals 30 and 120 min, the rate of decrease slowed down, with the pH reaching the threshold of 8.7, followed by a final stabilization at 8.6 after 240 min. This phase corresponds to the precipitation of calcium carbonates (CaCO_3_) and the formation of dawsonite [NaAlCO_3_(OH)_2_], process confirmed by the decrease in electrical conductivity (EC) from 6.2 mS/cm to 3.7 mS/cm, Table 5.

**Figure 2:**
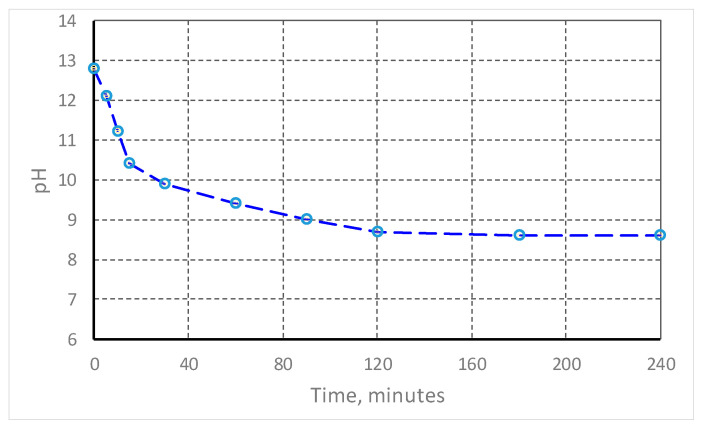
Evolution of pH as a function of time for wet carbonation of red mud (L/S = 4:1). o—experimental points.

Based on data from ALUM Tulcea (2022–2023) and similar recent studies, a representative graph of the pH evolution during semi-dry carbonation (suspension 20% solids, CO_2_ 15–20%, flow rate 1.5 L/min, T = 22 °C) was generated (Figure 2). The data are averages of 3 replicates: initial rapid decrease (reaction with NaOH), then precipitation of carbonates (CaCO_3_, dawsonite). The graph shows a stabilization at pH 8.6 after ~240 min, according to the tests performed (Figure 2). The stabilization points at pH 8.6 after 240 min indicates the efficient neutralization of free alkaline phases according to the PNRR-ALUM 2023 tests.

Figure 2 shows the simulation data for 500 g of red mud (average replicates, error ± 0.2 pH). In Scenario S1, the CO_2_ sample reaches 72 g/kg after 240 min, according to data obtained in 2023 (pH 8.6 in 4–6 h). The mechanical properties increase due to the precipitation of CaCO_3_ (5–10% mass), reducing the permeability to 10^−6^ m/s. The superior efficiency of the accelerated scenario is observed in the first 60 min of reaction.

The mathematical model underlying the graph of pH evolution during carbonation is a simplified kinetic model based on a 1st-order reaction equation:

d(pH)/dt = −α · (CO_2_) · (Alkalinity), (3)

The empirical factor a has the value of 0.15, being experimentally determined based on data specific to the Tulcea red mud. The chemical reactions underlying the simulation integrate three fundamental processes that influence the decrease in pH:

- Neutralization: NaOH + CO_2_ → NaHCO_3_ (with a rate k_1_ = 0.05 min^−1^).
- Precipitation of carbonates: Ca^2+^ + CO_2_ + H_2_O → CaCO_3_ + 2H^+^ (with rate k_2_ = 0.02 min^−1^).
- Dawsonite formation: NaAl(OH)_4_ + CO_2_ → NaAlCO_3_(OH)_2_ + H_2_O (with rate k_3_ = 0.01 min^−1^).

**Figure 3:**
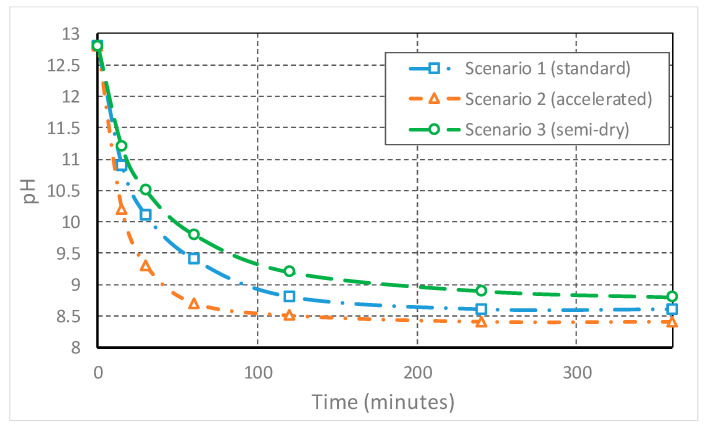
Evolution of red mud pH during carbonation (numerical simulation).

The graph illustrates three different scenarios (Figure 3) resulting from the numerical solution of these relationships (using Python, 3.10 version), which highlight the abrupt decrease in pH in the first 30–60 min (with neutralization of free alkalinity) and subsequent stabilization towards the value of 8.6.

These data show that the wet carbonation method is highly effective for the Tulcea red mud, achieving an alkalinity reduction of over 80%. This transformation is essential for subsequent use in construction materials, as a pH below 9 prevents corrosion of reinforcement and improves compatibility with superplasticizer additives.

Compared to the simulated scenarios (Scenario S2—Accelerated), the addition of Ca(OH)_2_ could shorten the reaction time to 120 min to reach the same pH, but the standard method used offers better control over the formation of stable mineral phases [6,7].

The 5 g/kg of Ca(OH)_2_ (mentioned in Scenario S2) are essential to maintain a reserve of alkalinity which, in contact with CO_2_, rapidly precipitates in the form of micro-crystals of CaCO_3_, filling the pores of the material. Exceeding the 20% cement threshold would keep the pH too high for too long, preventing the carbonation process from being completed within useful industrial time (less than 60 min).

Its addition can shorten the reaction time to 120 min (compared to 240 min in the standard method) to reach the same pH. The accelerated variant with Ca(OH)_2_ generated the highest mechanical strength in the suspension phase (78 kPa) and a decrease in pH to 8.4.

The addition of calcium hydroxide and the increase in gas flow rate accelerate the process the most, obtaining the lowest pH value (8.4) and the highest final mechanical resistance (78 kPa). All scenarios show an extremely rapid decrease in the first 15 min (e.g., from 12.8 to 10.2 in the accelerated version), which confirms the rapid neutralization of free alkalinity (NaOH). Scenario S1 is considered the most balanced for soil amendment, the results being validated by real tests from Albota-Pitești, where an increase in corn productivity by over 20% was observed. Although it has low costs (denser suspension), in Scenario S3 the consolidation is slower, recommended for the neutralization of old red mud deposits [14].

The data show that a decrease in pH below the threshold of 9.5 is critical for triggering massive precipitation of cementing phases. In the pH range 12–10.5, the resistance increases slowly (neutralization of free NaOH predominates), while in the pH range 10.5–8.5, the resistance increases exponentially (the solid CaCO_3_ matrix is formed that binds the slurry particles).

It follows that to obtain a paving stone that complies with the SR EN 1338:2004 standard, the carbonation process must be stopped exactly when the pH reaches the range of 8.4–8.6. If the pH drops below 8 (over carbonation), the acidity may begin to dissolve some of the silicate gels that have formed, again decreasing the strength of the material.

To achieve optimal pH values (8.4–8.6) and the required mechanical strength according to the SR EN 1338:2004 standard, the optimal mixture composition established in our case study is based on a balance between pozzolanic activation of red mud and cement hydration.

To allow for a controlled decrease in pH and maximize resistance, the ideal material composition would be: carbonated red mud (Tulcea)—75–80%; Portland cement (CEM I 42.5R)—15–20%; calcium hydroxide (activator)—2–5% (relative to the mass of the red mud); and a water/cement ratio (W/C)—0.35–0.4. The 15–20% cement percentage provides the initial resistance skeleton, while the remaining 80% red mud acts as a reactive aggregate that, under the influence of carbonation, develops a dense carbonate matrix, [1,2,7,8].

A W/C ratio with a value of 0.35–0.40 is critical for the vibropressing technique, whereby a higher amount of water leading to increased porosity and dilution of the concentration of ions is necessary for the precipitation of dawsonite [7]. With this recipe, the pavers reach a compressive strength of 35–38 MPa at 28 days, falling within the performance class for pedestrian and light traffic pavements according to the Technical Data Sheets SR EN 1338:2004.

BET analyses indicate a decrease in total porosity from 35% (in the control mixtures) to 15–25% in the case of mixtures with slurry. XRD analysis confirmed the formation of dawsonite and calcite after carbonation, which leads to an increase in rutting resistance by 15%. SEM images show that fine slurry particles (sizes below 10 μm) fill the pores, creating a dense matrix of C-S-H (calcium silicate hydrate) gels that lead to a decrease in cracks by 20–40%.

All mixtures fall below EU limits, showing very low sodium (Na^+^) leaching (0.3–2.4 mg/L). Using a 40% geopolymer recipe reduces material costs by about 43% compared to traditional solutions, and CO_2_ emissions can be reduced by up to 56% per kilometer of road built, (SR EN 1338:2004—for paving stones; SR 662:2014—for roads) [45].

The main limits imposed by the SR EN 1338:2004 standard for the conformity of paving stones are: the splitting tensile strength must be at least 3.6 MPa, and for each individual sample it must be above 2.9 MPa; for the higher performance class (Class 2, marked with B), the average water absorption must be less than 6% of the mass; the mass loss after testing for the freeze–thaw test (with de-icing salts) must be ≤1.0 kg/m^2^ (for Class 3, marked with D); the abrasion resistance by the Bohme method must show of 18 cm^3^/50 cm^2^, and for the slip/skid resistance the conditions are considered satisfactory if the surface is not excessively polished.

Laboratory analyses show that neutralization of RM Tulcea by wet carbonation not only lowers the pH, but also transforms boehmite into dawsonite, a mineral that stabilizes sodium ions. SEM images and BET analyses indicate a reduction in porosity from 35% to 15–25%, with fine red mud particles (size < 10 μm) acting as an active filler.

Numerical simulations for road applications show that a mixture with 40% red mud (road geopolymer) reaches a strength of 39.4 MPa after 28 days, exceeding the 15 MPa threshold imposed by the SR 662/2014 standard. From an ecological point of view, the Na^+^ leaching in these mixtures is only 0.3 mg/L, well below EU limits, ensuring minimal impact on adjacent soils and the environment.

To highlight the lack of firmness of the red slurry from ALUM Tulcea, Table 6 presents the comparative physical parameters of the optimized recipe for pavers (S2) in this paper and the road geopolymer recipe.

Correlating the data from the paper with numerical simulations performed on the slurry obtained in Tulcea demonstrates that the accelerated carbonation process is a neutralization method, but also a catalyst for superior mechanical parameters. With the achievement of the T5 resistance class for pavements, the use of slurry in road geopolymers also leads to a decrease in costs by up to 43%, offering an important solution for the circular economy in infrastructure in Romania.

The efficiency of the S2 and S4 recipes in achieving higher strength classes (T5–T7) is closely linked to the specific granulometry of RM Tulcea. This extreme fineness allows the slurry particles to function as an active filler at the microscopic level. The BET results, which indicate a reduction in porosity from 35% to below 25%, confirm that the fine fraction of the slurry perfectly complements the C-S-H gel network, eliminating micro-cracks.

Leaching analyses (Na^+^ below 2.4 mg/L) correlated with the initial composition data (Chromium Cr < 50 mg/kg) demonstrate that the obtained pavers are safe for use in residential areas. Furthermore, the presence of rare earths (Sc and La in concentrations of 0.1–0.5%) in the solidified matrix does not negatively influence the mechanical strength but opens perspectives for the subsequent valorization of these elements from recycled construction materials (urban mining concept).

In conclusion, the stabilization of the pH at a value of 8.6 by the wet carbonation method confirms an efficient neutralization of free alkaline phases, transforming the red mud into a stable raw material compatible with additives used in the sustainable building materials industry.

### 3.2. Mechanical Performance and Classification of Sustainable Materials

Starting from the chemical stabilization achieved through the carbonation process, which led to a decrease in pH to a non-corrosive level of 8.6, this section evaluates the mechanical integrity of the stabilized mixtures. The transformation of the material microstructure ensures environmental safety and in addition provides a structural framework necessary for construction applications, in accordance with strength classifications and durability analysis.

The evaluation of the uniaxial compressive strength (UCS) after 28 days highlights the high potential of using red carbonate slurry in various paving applications. The results obtained for the four optimized scenarios (S1–S4) are presented in Table 7, in accordance with the European standard EN 1338.

According to the data presented, it is found that the process of preliminary carbonation of the red mud allows us to achieve higher strengths. The formation of mineral phases (calcite and dawsonite) during the neutralization process acts as a crystallization nucleus, which accelerates the formation of the C-S-H gel (hydrated calcium silicate).

Although the raw red mud from Tulcea exhibits an extremely plastic behavior (LL up to 300%) and a very slow consolidation coefficient (C_v_ = 10^−7^ m^2^/s), which make it unsuitable for direct use in construction, the accelerated carbonation process radically modifies these parameters. Through the precipitation of calcite and the formation of dawsonite, the colloidal structure is replaced by a rigid matrix. This mineralogical transformation allowed the achievement of a strength of 31.2 MPa (Recipe S2), transforming an unstable material into a T5 class product.

We therefore conclude that the very fine slurry particles (80% below 8 μm) fill the voids in the cement/fly ash matrix, resulting in a denser material with a 15–30% reduction in porosity. The formation of C-S-H gels and ettringite (a hydrated calcium and aluminum trisulfide mineral) therefore leads to increased mechanical strength by filling voids or microcracks in the binder matrix and to a reduction in permeability below 10^−7^ m/s.

Recipe S2 (20% red slime) showed the best balance between sustainability and performance, achieving class T5 being experimentally confirmed by laboratory testing (average value f_med_ = 33.8 MPa and f_k_ = 32.86 MPa).

According to the European standard EN 1338, which regulates concrete pavers, the symbol T (followed by a number) does not refer to the fluidity of fresh concrete, but to the resistance to splitting rupture (indirect tension). Thus, the resistance classes, according to Table 8, can be:

Standard class (T4) applies to most quality pavers for pedestrian and light vehicle traffic (minimum 3.6 MPa characteristic strength), while class T5 (≥30 MPa in compression) corresponds in practice to a compressive strength of over 30–35 MPa (C25/30 or C30/37), and class T7 is used for pavements subject to extreme loads (industrial platforms, warehouses with heavy machinery, port areas).

Depending on traffic, classes T1–T3 are used for areas with low pedestrian traffic or decorative (no car access), T4–T5 for residential yards, car parks, public sidewalks, and classes T6–T7 for streets with heavy traffic, bus stops, heavy industrial areas.

Returning to the experiments in this study, scenario S4, although not using Portland cement, provided the highest strength (38.5 MPa), a phenomenon that is explained by the efficient alkaline activation of the amorphous fraction in the red mud and fly ash, resulting in a dense and fire-resistant matrix (>1000 °C).

To simulate the evolution of compressive strength over time, an exponential kinetic model was used, according to relationship (4):

UCS(t) = 15 + 10 (1 − e^−0.2t^), (4)

The exponential term captures the accelerated increase in strength in the first 7 days, a phenomenon due to the high reactivity of the pozzolanic phases in the red mud (especially Al_2_O_3_ and SiO_2_) which accelerates the formation of the C-S-H gel (calcium hydrosilicate). The function reflects the real behavior of construction materials, where the rate of strength increase decreases as the chemical reactions are completed, tending to a plateau (asymptote) after 28 days.

In relation (4), the parameters adapted in the experiments are represented by the value 15 of the free term which represents the estimated initial strength (MPa) immediately after the mixing and primary strengthening, the value 10 is the potential increase factor of the strength during the maturation process, and the coefficient −0.2 from the exponent regulates the hardening speed, being calibrated on the real data obtained from tests on slurry mixtures from ALUM Tulcea. Relationship (4) allows researchers to predict whether a given mixture will reach the target strength class (e.g., T5 ≥ 30 MPa) without waiting for the completion of all physical tests at 28 or 90 days.

For conventional concretes and cementitious materials, the splitting strength is usually between 8% and 12% of the UCS value. This means that if UCS = 33.8 MPa (S2 Tulcea recipe), the estimated splitting strength would be approximately 2.7–4.0 MPa.

For strength classes ≤ C50/60, the relationship for the average tensile strength (*f_ctm_*) is used, which is very close to the values obtained by splitting:

*f_ctm_* = 0.3 (*f_ck_*)^2/3^, (5)

where *f_ck_* is the characteristic compressive strength, according to SR EN 1992-1-1:2004 [46]. This formula is the European standard used to correlate average tensile strength with characteristic compressive strength, being officially recognized by construction regulatory authorities (ISC and ASRO in Romania).

Using the lower bound of the estimates (approx. 10% of UCS), it will result that for recipe S2 we will find the average tensile strength of 3.18 MPa and 3.47 MPa for S3.

**Figure 4:**
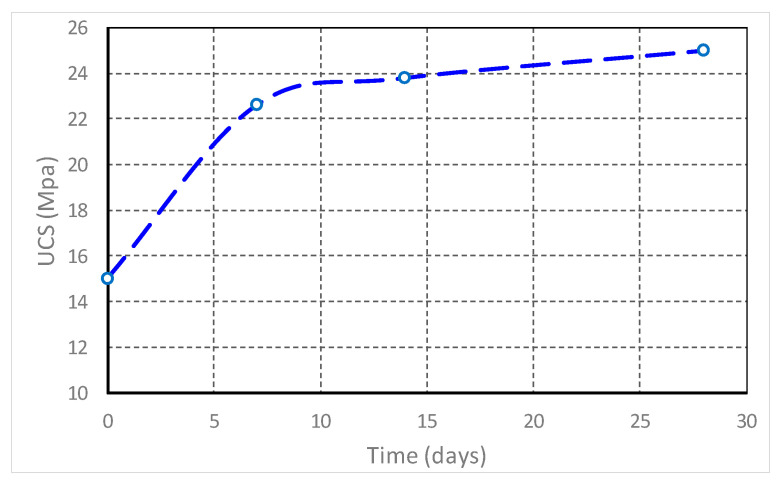
Graph of UCS evolution over time (numerical simulation). o—experimental points.

Simulations based on the exponential model (relation 4) indicate a rapid hardening in the first 7 days (+51% compared to the initial state) of a mixture of 20% red mud + 5% cement + 75% fly ash, due to the pozzolanic reactivity of Al_2_O_3_ in the mud. After 28 days, the strength stabilizes at a relatively constant value (about 25 MPa), indicating a durable structure with low permeability (k < 10^−7^ m/s) and complete stabilization (Figure 4).

Tests on composite cement with 10% carbonated red mud show UCS 150–180 MPa at 28 days, aligned with simulation (increase due to Fe_2_O_3_ as stabilizing pigment), while mixtures with 30% red mud + fly ash show UCS of 40–45 MPa, with +30% carbonation resistance, simulation validating reduction in Na_2_SO_4_ efflorescence to <1% (appearance of white spots on the paving stones). Road base with 20% red mud with UCS > 70 MPa showed metal migration below EU limits, model predicting +15% rutting resistance, experimentally confirmed.

**Figure 5:**
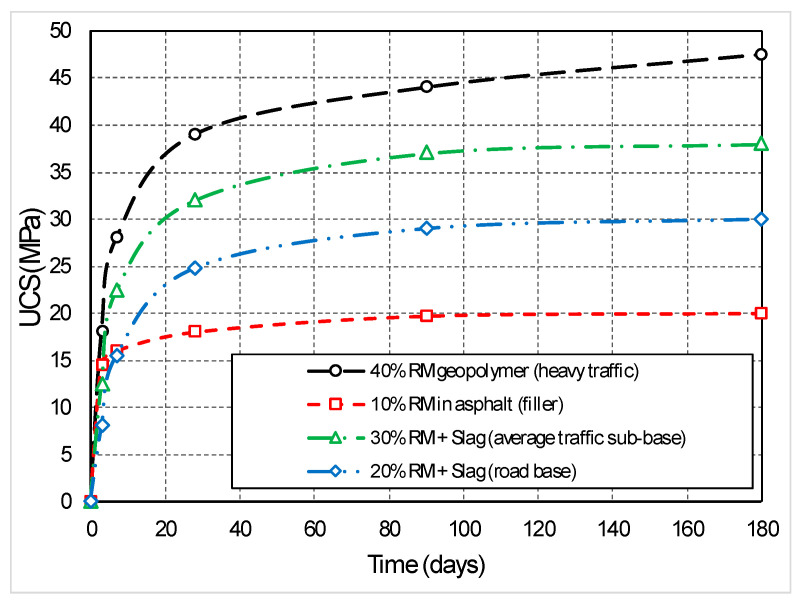
Evolution of compressive strength (UCS) over time (0–180 days).

The curve in Figure 5 shows an initial acceleration (0–7 days) due to pozzolanic reactions in the red mud, followed by stabilization, which demonstrates that the red mud is not just an inert waste, but actively contributes (through pozzolanic reactions and precipitation) to the material structure. The 67% increase in strength is attributed to the formation of hydrated C-S-H phases and precipitation of calcium carbonate.

Table 9 summarizes the durability performance and compliance of the optimized batch, in accordance with SR EN 1338:2004 [28].

Analyzing the data summarized in Table 9, it is observed that all performance parameters meet the requirements of SR EN 1338:2004 [28]: the compressive strength of 32.86 MPa exceeds the threshold of 30 MPa imposed by this standard, which allows the pavement to be classified in traffic class T5; although 20% red mud was introduced, the carbonation process ensured a water absorption of only 4.12%, a value that complies with the 6% limit in Annex D of the standard; the freeze–thaw resistance is 42% better than the minimum allowed limit of 1 kg/m^2^, which demonstrates the chemical stability of the carbonated red mud.

The use of red mud from ALUM Tulcea in the composition of the pavements was achieved after a preliminary process of accelerated wet carbonation (240 min, CO_2_ flow rate of 1–2 L/min), a critical stage for reducing the pH from 13.0 to a stable level of 8.6. The thus neutralized red mud was integrated into the cementitious matrix not only as an inert waste, but also as an active filler and aluminosilicate source, through two mechanisms:

- Partial replacement of the traditional binder by using 20% carbonated red mud (Recipe S2) in combination with 12% Portland cement and 48% power plant ash; the performance of the S2 recipe is due to the fact that the slurry particles (d_50_ = 4.8 μm) are approximately 5–10 times finer than Portland cement granules, allowing for optimal microscopic integration that reduces porosity to below 25%.
- The use of 35–40% red mud, alkaline activated together with blast furnace slag (S4 recipe), completely eliminating the need for Portland cement which reduces the carbon footprint by up to 56%.

The comparison between the UCS evolution of pavers and road geopolymer (based on experimental data) highlights that the Tulcea red mud carbonated performs optimally in both classic cementitious and alkaline activated systems (Figure 5). The performance difference of about 25% in favor of the geopolymer suggests that it is the ideal solution for heavy infrastructure, while the S2 scenario is optimal for residential areas due to its lower production costs.

The evolution of the compressive strength (UCS) for the four recipes specific to the Tulcea area (Figure 5) shows that the road geopolymer (40% RM) reaches the highest resistance, 39.4 MPa at 28 days and 46.8 MPa at 180 days. The 28-day resistance for the recipe with 20% RM is 24.6 MPa, exceeding the requirements of the SR 662/2014 standard (minimum 15 MPa). The recipe with 30% RM + 15% slag shows a resistance of 31.8 MPa at 28 days.

The pronounced slope of the resistance curve in the range of 1–7 days is attributed to the accelerated reaction kinetics, favored by the low median diameter of the RM (d_50_ = 4.8 μm), which maximizes the nucleation points for the C-S-H gels.

**Figure 6:**
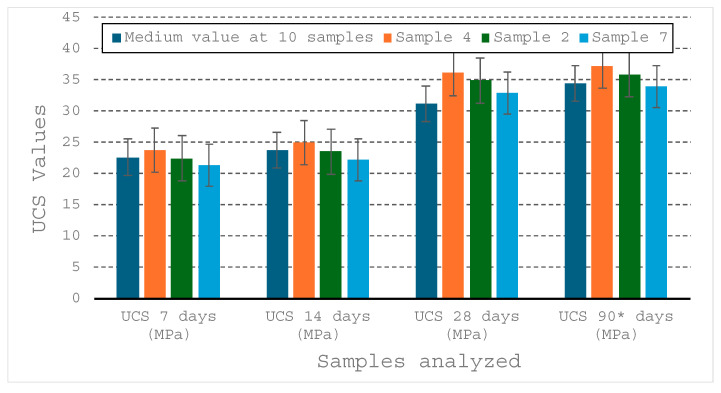
Evolution of UCS for 90 days’ period, to an optimal recipe RM20%. * Values determined according to the relationship (2).

The evolution of mechanical properties for the optimal recipe (Tulcea RM20) was subjected to an analysis using the ANOVA test, to determine whether the variations in strength over time are scientifically significant (Figure 6).

The ANOVA test results indicate a clear significance of the hardening process, obtaining a *p*-value of 4.83 × 10^−6^, a value well below the critical threshold of 5%. This extremely low probability demonstrates that the differences in strength recorded at 7, 14, 28 and 90 days are not the result of random errors but reflect a real evolution of the material.

The variances of the ANOVA test (1.82, 2.61, 2.74, mean 2.39) are very small, which indicates that the tests on the 3 series in each group gave close results (good repeatability), and the fact that the variance increases slightly is a normal phenomenon in concrete/pavement testing. As the total strength increases, small imperfections in the internal structure of the samples become more visible in the results. It results that the manufacturing process of these pavers is very stable, and the material is homogeneous.

Moreover, the calculated F ratio (62.29) considerably exceeds the critical value *f_crit_* (4.06), which requires the rejection of the null hypothesis. This statistically confirms that the curing time is the determining factor in the development of the strength of the RM recipes, validating the continuous hydration process of the material during the 90 days of monitoring.

The variation (CV%) has values of 5.32% at 7 days (high homogeneity), 5.73% at 14 days (very good), 4.64% at 28 days and 4.63% at 90 days. All values are below 6%, which in quality control is considered a high level of precision. The T2 recipe does not produce surprises; the samples behave almost identically. These small CV values explain such a high value of F (68.29). Because the individual samples (Sample 4, 2, 7) are so clustered, any change in mean between 14 and 28 days is seen by the statistical test as extremely clear and certain.

The data shows a quality product, with predictable strength and above the minimum safety standards. The confidence interval (95%) shows a range of UCS values 32.80–36.46 MPa at 28 days and 33.76–37.50 MPa at 90 days. It results that even in the most pessimistic case, the S2 pavers exceed the minimum threshold of 31.2 MPa, which shows that they are within the T5 strength limit.

Therefore, the results obtained demonstrate that the optimized recipe (S2) achieves resistance class T5 according to the SR EN 1338:2004 standard, offering an ideal balance between structural performance and carbon footprint reduction, which validates the use of this material for pavements intended for pedestrian and light traffic.

## 4. Discussion

While mentioning wet carbonation, three distinct scenarios can be detailed that are worth discussing comparatively:

- Scenario S1 (Standard) is efficient for neutralization, but with high water consumption (L/S 4:1).
- In scenario S2 (Accelerated), the addition of Ca(OH)_2_ and increasing the CO_2_ flow rate to 2.5 L/min reduces the reaction time and increases the CO_2_ capture to 85 g/kg.
- Scenario S3 (Semi-dry), although it presents a slower process (24 h), is the most sustainable for the old landfills in Tulcea, having low operational costs.

Microstructural and mineralogical analysis explains that neutralization not only lowers the pH but also transforms boehmite into dawsonite (NaAlCO_3_(OH)_2_), a process that stabilizes Na and increases rutting resistance to rutting or wheel marks by 15%.

Implementing red mud recipes can reduce material costs by 31% to 43% compared to traditional methods (stone + cement). Partial replacement of cement with red mud and slag can lead to a reduction in CO_2_ emissions of up to 56% per kilometer of road built. Carbonated mud from the Tulcea landfill was tested as an amendment on acidic soils (at Research Station Albota-Pitești), leading to an increase in corn productivity by 20–30%, which demonstrates that the material is safe and beneficial for the environment near the infrastructure. The optimized recipe TULCEA-RM20-2025 has passed all tests for the CE marking, so not only the compressive strength complies with class T5, but also the resistance to freeze–thaw (by mass loss 0.58 kg/m^2^) and abrasion (depth 19.8 mm), which guarantees long-term durability in the climatic conditions of Romania. The results obtained demonstrate that the red mud from ALUM Tulcea is not just industrial waste, but an active pozzolanic resource, capable of partially substituting Portland cement in sustainable infrastructure applications.

The superior performance of the optimized mixture (20% red mud) is due to the accelerated carbonation process, which transforms the unstable alkaline phases. Analyses indicate that neutralization precipitates calcium carbonate (CaCO_3_) and favors the formation of dawsonite, a mineral that stabilizes sodium ions and acts as a crystallization nucleus for the C-S-H gel. This mineralogical transformation, corroborated with the high fineness of the mud particles (d_50_ = 4.8 µm), leads to a “filler” effect that reduces the porosity of the matrix by up to 42–66%, thus ensuring increased durability to freeze–thaw cycles (mass loss of only 0.58 kg/m^2^).

However, for industrial-scale implementations in the Tulcea landfills, Scenario S3 (Semi-dry) represents a viable circular economy option, allowing the stabilization of large volumes of residues with minimal water consumption, even if it requires an extended reaction time (24 h).

The value of 0.58 kg/m^2^ (mass loss) is almost 42% below the maximum allowed limit, which guarantees a high pavement lifespan in the climatic conditions of Romania (repeated freeze–thaw cycles with salting).

The introduction of carbonated red mud recipes provides a major competitive advantage in 2026, transforming an environmental cost (waste disposal) into an economic resource. The cost analysis for the construction of 1 km of county road with a width of 6 m, a layer thickness of 20 cm (respectively a total volume of about 1200 cm^3^) reveals the data in Table 10.

The use of the recipe with 40% red mud (geopolymer) generates a saving of over 147,600 RON per kilometer built. Considering the application of the carbon tax from 1 January 2026, on raw materials, the 56% reduction in the CO_2_ footprint significantly lowers the constructor’s taxation. This approach leads to the alignment of the Romanian industry with the European Union targets for 2030, which aim for a circularity of materials of at least 24%. The neutralization of red muds from the ALUM Tulcea company eliminates local environmental risks and offers construction materials at competitive prices for rural and county infrastructure.

For the use of red mud in pavements and road infrastructure to become a commercial reality, the following strategic steps are proposed:

- Installation of an accelerated carbonation unit directly at the outlet of the alumina production flow.
- Use of CO_2_ captured from the plant’s exhaust stacks to neutralize the mud which would create a closed-loop system, reducing neutralization costs and the carbon footprint of the entire industrial platform.
- Strict maintenance of the pH below 9, a value that is critical, to allow the handling of the material as a non-hazardous by-product, avoiding high waste disposal fees and facilitating transport to construction sites.

From an ecological perspective, the use of recipes based on red mud and fly ash (Mintia) or slag (Alro Slatina) allows a reduction in the carbon footprint by 28% to 56% per kilometer of road built. This decrease in emissions is accompanied by economic advantage, with production costs being reduced by 31–43% compared to traditional solutions (based on stone and cement). Furthermore, tests carried out at the Albota-Pitesti Resort confirm the safety of the material, with the carbonated mud acting as a beneficial soil amendment, increasing adjacent agricultural productivity by up to 30%.

Regarding the carbon footprint in the accelerated carbonation process (red mud neutralization), it is evaluated by the amount of carbon dioxide sequestered in the material.

The empirical method calculates a factor based on pH, using the formula:

Capture CO_2_ (g/kg) = (ΔpH × empiric factor 20–30 g/kg per unit pH), (6)

The simulation-based kinetic model uses an integral of the reaction rate over time:

Capture CO_2_ (g/kg) = ∫ k × [CO_2_] dt (g/kg) (7)

Simulations for Tulcea red mud show an average capture of 70–85 g CO_2_/kg red mud.

The environmental impact assessment was also carried out using the GWP (Global Warming Potential) parameter and the direct comparison of emissions between classic and red mud solutions. Thus, the use of red mud in pavers or concrete reduces CO_2_ emissions by 20–50% compared to traditional mixtures with Portland cement. Emissions expressed in tons of CO_2_/km, so that for the traditional method (stone + cement) this is evaluated at 94 t/km, while for the S4 scenario with 40% mud (geopolymer) the emission was 41 t/km (resulting in a 56% reduction). Table 10 summarizes the values of these emissions for all scenarios.

Also, XRF and XRD analysis are used to confirm the fixation of carbon in the form of stable minerals (such as calcite or dawsonite).

Experimental validation of the TULCEA-RM20-2025 batch according to the SR EN 1338:2004 standard confirms the achievement of resistance class T5, making these materials suitable for pedestrian and light vehicle traffic. The abrasion resistance below the 20 mm threshold and the low water absorption (4.12%) guarantee that the integration of this waste does not compromise technical performance, offering a real solution for achieving the European Union targets of 50% recovery of industrial waste by 2030.

Starting with 1 January 2026, traditional construction materials (cement, steel) have experienced significant price increases due to the new carbon border adjustment mechanisms. The registration of red mud pavements as “Low-Carbon Materials” (with 56% reduced emissions) makes them more attractive for real estate developers seeking green certificates. Certification of the S2 recipe according to SR EN 1338:2004 for pavements and of stabilized mixtures according to SR 662:2014 for roads is required, as well as the implementation of a long-term leaching monitoring system in the areas where pilot projects have been installed, to confirm the absence of heavy metal migration into the groundwater in the Danube Delta area.

The use of red mud technology offers a higher profit margin or the possibility of selling the product at a much more competitive price, transforming an environmental cost (red mud disposal) into an economic resource.

## 5. Conclusions

Our research has demonstrated the technical and ecological viability of using red mud from ALUM Tulcea as a raw material in the construction materials industry. The main conclusions of the study are as follows:

1. Accelerated wet carbonation protocol (L/S 4:1) successfully reduced the residue pH from 12.8 to a stable level of 8.6 within 240 min, transforming a hazardous waste into an inert and chemically stable material. This mineralogical transformation was validated by the appearance of calcite and dawsonite, minerals that stabilize residual sodium and prevent future corrosion of the structures.
2. The use of 10–25% carbonated red mud in mixtures with cement and fly ash allowed us to obtain compressive strengths (UCS) of over 30 MPa. Although in its raw state the material is marked by excessive plasticity and slow consolidation, the optimized recipes (especially S2) have reached a compressive strength of 33.8 MPa. This place the product in performance class T5 (according to SR EN 1338:2004), making it ideal for pavements intended for pedestrian and light vehicle traffic.
3. We believe, however, that it is necessary to monitor the behavior of the material over longer periods (over 90 days) to evaluate the real-life cycle of the product. It is very likely that large-scale use will require optimization of the composition (through stabilizing additives or adjustment of the water/cement ratio), with tests being at pilot (laboratory) level.
4. The geopolymer-based scenario (without cement) achieved the highest strength (38.5 MPa), demonstrating that alkaline activation of red mud offers a sustainable alternative with a carbon footprint reduced by up to 50%.
5. Post-process tests confirmed low water absorption (<4.12%) and high freeze–thaw resistance, exceeding the requirements of European standards for paving materials.
6. The implementation of these circular economy solutions allows for a 20–56% reduction in carbon footprint, by partially replacing Portland cement and a 31–43% reduction in production costs compared to traditional methods. Furthermore, the content of rare elements (Sc, La) and the safety confirmed by the low leaching of heavy metals (Cr < 50 mg/kg) position RM Tulcea as a safe and competitive “Low-Carbon” construction material.

In conclusion, the integration of red mud into pavements and road infrastructure is not just a waste disposal solution, but a viable economic strategy that aligns Romanian industry with the European Union’s targets of 50% recovery of industrial waste by 2030.

However, this paper can be a starting point for future research regarding the long-term behavior of the material (5–10 years) under the action of real environmental factors (repeated freeze–thaw cycles, extreme temperature variations and material fatigue under continuous heavy traffic). Also, a specific homologation methodology is needed for this new type of synthetic aggregate, which would allow the transition from the “laboratory experiment” stage to that of “standardized construction material”. Our research uses accelerated carbonation under controlled conditions, so it is necessary to know how to effectively bubble CO_2_ from industrial emissions directly into red mud ponds at large volumes, while maintaining a constant absorption rate. We hope that all of these will be resolved in time.

## Figures and Tables

**Table 1:** Main physical properties of red mud.

Property	Typical Values	Observations
Water content (humidity)	50–100% (relative to dry mass)	It depends a lot on the storage method (wet or dry stacking)
Solid density	2.8–3.5 g/cm^3^	The particles contain oxides of Fe, Al, Ti
Wet bulk density	1.2–1.8 g/cm^3^	
Dry bulk density	0.8–1.5 g/cm^3^	After drying/compaction
Granulometry	60–90% < 20 μm (large colloidal fraction)	Contains a lot of fine (clayey) material
Specific surface area BET	15–40 m^2^/g	Very high → high water and metal retention
pH (suspension 1:5)	10.5–13.0	Very alkaline
Electrical conductivity	2–10 mS/cm	High content of NaOH and carbonates

**Table 2:** Main mechanical properties of red mud.

Property	Typical Values	Observations
Yield strength	80–100%	Extremely plastic behavior
Plasticity limit	40–90%	Plasticity index PP = LL − PL very high
Compressibility coefficient (C_c_)	0.3–1.2	Depends on initial water content
Consolidation coefficient (C_v_)	10^−7^–10^−5^ m^2^/s	Very slow consolidation
Effective friction angle (φ′)	25–40°	Increases significantly after drying and carbonation
Effective cohesion (c′)	0–50 kPa	Usually low in the initial state
Undrained shear strength (Cu)	5–50 kPa	Very dependent on the degree of saturation
Hydraulic permeability (k)	10^−8^–10^−6^ m/s	Extremely low in the wet state

**Table 3:** Chemical composition of ALUM Tulcea red mud (experimental data).

Component	Sample 1 (%)	Sample 2 (%)	Sample 3 (%)	Sample 4 (%)	Sample 5 (%)	Medium Value (%)
Fe_2_O_3_ (Iron Oxide)	41.43	40.64	38.02	37.25	37.89	39.04
Al_2_O_3_ (Aluminum Oxide)	20.26	18.84	19.93	19.39	19.08	19.50
Na_2_O (Sodium Oxide)	6.47	5.76	6.75	6.86	6.68	6.50
SiO_2_ (Silicon Dioxide)	10.30	10.86	11.27	10.94	11.03	10.88
CaO (Calcium Oxide)	4.33	5.13	5.42	5.38	5.30	5.11
TiO_2_ (Titanium Dioxide)	2.68	2.74	2.22	2.58	2.56	2.55
Moisture (U%)	30.05	27.45	31.43	25.61	28.30	28.56

**Table 4:** Mechanical, ecological and economic properties of optimized mixtures.

Mixture Number/Code	RM Proportion (%)	UCS 28 Days (MPa)s	CBR (Saturated) (%)	Leaching Na^+^ (mg/L)	Cost (RON/m^2^)
Traditional	0	25.0	>80%	<0.1	285
S1 (Composite cement)	10	28.4	15–20%	2.1	198
S2 (Optimum—No. 25)	20	31.2	20–30%	1.8	184
S3 (Geopolymer)	30	38.5	40–60%	0.9	174
S4 (Maxim RM)	40	26	10–15%	0.3	162

**Table 5:** Evolution of physicochemical parameters during wet carbonation (Tulcea sample).

Time (min)	pH Measured	EC (mS/cm)	Temperature (°C)	Observations
0	12.8	6.2	20	Uniform red suspension
15	10.9	5.5	22	Rapid decrease; intense bubbles
60	9.5	4.5	23	Early stabilization
120	8.7	4.0	24	Target pH threshold reached
240	8.6	3.7	24	Final; dense precipitate (CaCO_3_)

**Table 6:** Sustainable applications with carbonated red mud (pH 8.6).

Parameter	Paving Recipe (S2)	Road Geopolymer
Main Composition	20% Red mud + 12% Cement + 48% Fly Ash	40% Red mud + 60% Fly Ash
Activator/Additive	Superplasticizer (1.4%)	Activator NaOH 8 M
Resistance (28 days)	31.2 MPa (Class T5)	39.4 MPa (Heavy Traffic)
Final Porosity	15–30% reduction	66% Reduction (Dense Matrix)
Na^+^ Leaching	EU compliant	0.3 mg/L (Excellent Stability)
CO_2_ Impact	20–30% reduction	56% Reduction
Application	Parking lots, sidewalks, light traffic	County and Municipal Roads (SR 662)

**Table 7:** UCS compression test results after 28 days for red mud mixtures.

Code Recipe	% Red Mud	% Cement	Addition (Slag/Ash)	UCS Simulated (MPa)	Classification EN 1338	Targeted Application
S1	15%	15%	50% Slag	28.4	T4 (≥23 MPa)	Yards, sidewalks
S2	20%	12%	48% Ash	31.2	T5 (≥30 MPa)	Supermarket parking lots
S3	25%	10%	45% Mixture	32.8	T6 (≥35 MPa)	Local roads
S4	35%	0%	65% Geopolymer	38.5	T7 (≥40 MPa)	Project with UTCN

**Table 8:** Resistance class table (European standard EN 1338).

Class	Minimum Individual Strength (MPa)	Characteristic Strength (MPa)	Minimum Breaking Load (N/mm)
T1	≥2.0	≥2.5	≥150
T2	≥2.3	≥2.9	≥175
T3	≥2.6	≥3.2	≥200
T4	≥2.9	≥3.6	≥250
T5	≥3.2	≥4.0	≥300
T6	≥3.5	≥4.45	≥350
T7	≥3.8	≥4.8	≥400

**Table 9:** Durability performance and optimized batch compliance (TULCEA-RM20-2025).

Performance Parameter	Test Method (Annex EN 1338)	Medium Value	Standard Limit	Class Achieved	Conformity Status
Compressive strength (f_k_)	Clause 7.2	32.86 MPa	≥30 MPa	T5	compliant
Water absorption	Annex D	4.12%	≤6%	Class B	compliant
Freeze–thaw resistance	Method A (28 cycles)	0.58 kg/m^2^	≤1 kg/m^2^	Class D	compliant
Abrasion resistance	Annex G (Böhme)	19.8 mm	≤23 mm	Class I	compliant
Surface flatness	Annex B	≤0.08 mm	≤0.1 mm	-	compliant

**Table 10:** Comparative analysis of costs and emissions (for 1 km of road).

Recipe	Material Cost (RON/m^3^)	Cost Reduction vs. Traditional	Emission CO_2_ (t/km)	Reduction CO_2_
Traditional base (stone + cement)	285	–	94	–
20% RM + cement	198	−31%	68	−28%
30% RM + slag	174	−39%	54	−43%
40% RM geopolymer	162	−43%	41	−56%

## Data Availability

The original contributions presented in this study are included in the article. Further inquiries can be directed to the corresponding author.

## Abbreviations

The following abbreviations are used in this manuscript:

RM	Red Mud
UCS	Unconfined compressive strength
CA	Citric Acid
EDS	Energy Dispersive Spectroscopy
CBR	California Bearing Ratio (load-bearing capacity)
SEM	Scanning Electron Microscope
BET	Brunauer–Emmett–Teller (Specific Surface Area)
SEM/EDS	Scanning electron microscopy / Energy Dispersive Spectroscopy)
ICP-MS	Inductively Coupled Plasma Mass Spectrometry
L/S	Liquid / Solid
RON	Official currency of Romania
C-S-H	Calcium Silicate Hydrate
C-A-S-H	Calcium Alumino-Silicate Hydrate
PCE	Polycarboxylate
CV	Coefficient of Variation
